# The spectrum of neurological disease associated with Zika and chikungunya viruses in adults in Rio de Janeiro, Brazil: A case series

**DOI:** 10.1371/journal.pntd.0006212

**Published:** 2018-02-12

**Authors:** Ravi Mehta, Cristiane Nascimento Soares, Raquel Medialdea-Carrera, Mark Ellul, Marcus Tulius Texeira da Silva, Anna Rosala-Hallas, Marcia Rodrigues Jardim, Girvan Burnside, Luciana Pamplona, Maneesh Bhojak, Radhika Manohar, Gabriel Amorelli Medeiros da Silva, Marcus Vinicius Adriano, Patricia Brasil, Rita Maria Ribeiro Nogueira, Carolina Cardoso Dos Santos, Lance Turtle, Patricia Carvalho de Sequeira, David W. Brown, Michael J. Griffiths, Ana Maria Bispo de Filippis, Tom Solomon

**Affiliations:** 1 National Institute for Health Research Health Protection Research Unit in Emerging and Zoonotic Infections, University of Liverpool, Liverpool, United Kingdom; 2 Institute of Infection and Global Health, University of Liverpool, Liverpool, United Kingdom; 3 Department of Neurology, Hospital Federal dos Servidores do Estado, Rio de Janeiro, Brazil; 4 Department of Neurology, Walton Centre NHS Foundation Trust, Liverpool, United Kingdom; 5 Laboratório de Pesquisa em Neuroinfecção, Instituto Nacional de Infectologia Evandro Chagas, Rio de Janeiro, Brazil; 6 Department of Neurology, Hospital de Clínicas de Niterói, Niterói, Brazil; 7 Department of Biostatistics, Institute of Translational Medicine, University of Liverpool, Liverpool, United Kingdom; 8 Department of Neurology, Hospital Universitário Pedro Ernesto, Rio de Janeiro, Brazil; 9 Department of Neurology, Hospital Geral de Bonsucesso, Rio de Janeiro, Brazil; 10 Department of Neurology, Hospital Barra D'or, Rio de Janeiro, Brazil; 11 Laboratório de Pesquisa Clínica em Doenças Febris Agudas, Instituto Nacional de Infectologia Evandro Chagas, Rio de Janeiro, Brazil; 12 Flavivirus Reference Laboratory, Oswaldo Cruz Institute, Rio de Janeiro, Brazil; 13 Influenza Reference Laboratory, Oswaldo Cruz Institute, Rio de Janeiro, Brazil; 14 Virus Reference Department, National Infection Service, Public Health England, London, United Kingdom; 15 Department of Neurology, Alder Hey Children's NHS Foundation Trust, Liverpool, United Kingdom; University of Texas Medical Branch, UNITED STATES

## Abstract

**Background:**

During 2015–16 Brazil experienced the largest epidemic of Zika virus ever reported. This arthropod-borne virus (arbovirus) has been linked to Guillain-Barré syndrome (GBS) in adults but other neurological associations are uncertain. Chikungunya virus has caused outbreaks in Brazil since 2014 but associated neurological disease has rarely been reported here. We investigated adults with acute neurological disorders for Zika, chikungunya and dengue, another arbovirus circulating in Brazil.

**Methods:**

We studied adults who had developed a new neurological condition following suspected Zika virus infection between 1^st^ November 2015 and 1^st^ June 2016. Cerebrospinal fluid (CSF), serum, and urine were tested for evidence of Zika, chikungunya, and dengue viruses.

**Results:**

Of 35 patients studied, 22 had evidence of recent arboviral infection. Twelve had positive PCR or IgM for Zika, five of whom also had evidence for chikungunya, three for dengue, and one for all three viruses. Five of them presented with GBS; seven had presentations other than GBS, including meningoencephalitis, myelitis, radiculitis or combinations of these syndromes. Additionally, ten patients positive for chikungunya virus, two of whom also had evidence for dengue virus, presented with a similar range of neurological conditions.

**Conclusions:**

Zika virus is associated with a wide range of neurological manifestations, including central nervous system disease. Chikungunya virus appears to have an equally important association with neurological disease in Brazil, and many patients had dual infection. To understand fully the burden of Zika we must look beyond GBS, and also investigate for other co-circulating arboviruses, particularly chikungunya.

## Author summary

During 2015–16, Brazil experienced the largest outbreak of the mosquito-borne Zika virus ever reported and saw a subsequent increase in cases of Guillain-Barré syndrome (GBS), a disorder of the peripheral nervous system (nerves outside the brain and spinal cord) that can result in paralysis and sometimes death. In this assessment of adults presenting with suspected Zika virus-associated neurological disease in Rio de Janeiro, Brazil, we add to the growing body of evidence linking Zika to a wider range of neurological disease, including disease of the central nervous system (brain and spinal cord). We also show that many patients initially thought to have neurological disease associated with Zika virus were in fact infected with chikungunya virus, another arthropod-borne virus (arbovirus) that is also associated with a wide range of neurological disease. Importantly, many patients had evidence of infection with more than one virus. We discuss the challenges in diagnosing the infections and how different body fluid samples can be used to help facilitate this. Our results suggest that clinicians and public health officials must look beyond GBS if we are to understand fully the disease burden of Zika virus. In addition, we highlight the need to investigate patients with acute neurological syndromes for other co-circulating arboviruses, particularly chikungunya.

## Introduction

Zika virus is an arthropod-borne virus (arbovirus) first isolated in Uganda in 1947, which spread to cause large outbreaks in Micronesia in 2007, French Polynesia in 2014 and Latin America from 2015.[1] By December 2015, it had caused an estimated 0·4–1·3 million cases in Brazil alone.[2] Like the related dengue viruses, Zika is a flavivirus (genus *Flavivirus*, family *Flaviviridae*) that causes a fever-arthralgia-rash syndrome and is transmitted principally by *Aedes* mosquitoes. An apparent association between Zika virus and an increase in severe congenital disease and other neurological disorders, particularly Guillain-Barré syndrome (GBS),[3–6] prompted the World Health Organisation to declare Zika virus a public health emergency of international concern in February 2016.[7]

A carefully conducted case-control study of the French Polynesian outbreak showed an association between Zika virus infection and GBS,[4] although prior dengue exposure made interpretation of the virology results challenging because of serological cross reactivity between flaviviruses.[8] Dengue, like other flaviviruses including Japanese encephalitis and West Nile viruses, can also cause both peripheral and central nervous system (CNS) disease.[9] Secondary dengue infections are associated with more severe dengue disease, and some have postulated that prior dengue may predispose to more severe Zika infection. More recently, a study from Colombia has shown a strong temporal association between GBS and Zika virus, with viral RNA detected in samples from 17 patients.[5] A few case reports have described Zika virus-associated myelitis,[10] encephalitis,[11, 12] meningoencephalitis,[13] acute disseminated encephalomyelitis,[14] Miller-Fisher syndrome,[15] and myasthenia gravis,[16] suggesting that the spectrum of neurological disease may be broader than initially thought. A preliminary epidemiological report from the French Polynesian outbreak indicated a possible increase in other neurological manifestations associated with Zika virus, but gave few details.[17]

Chikungunya is another arbovirus also identified in Africa in the 1950s that has spread to cause epidemics in the tropics in recent years.[18] It was first reported in Latin America in 2013 and has caused large outbreaks in Brazil in the last two years, with over 260,000 suspected cases in 2016.[19] Like Zika and dengue viruses, it is transmitted by *Aedes* mosquitoes and leads to a fever-arthralgia-rash syndrome; it also occasionally presents with neurological disease, including GBS, encephalitis and myelitis,[20] although there are few such reports from South America.[21] Because chikungunya is an alphavirus (genus *Alphavirus*, family *Togaviridae*) there is no serological cross reactivity with the flaviviruses, making diagnosis more straightforward. Although chikungunya virus co-circulates in many Zika-affected areas, including Colombia and Brazil, and can cause neurological disease, its role has not been assessed.

To assess the spectrum of neurological disease associated with Zika virus, we studied adults in Rio de Janeiro with acute neurological syndromes following suspected Zika virus infection. Given their similarities and co-circulation, we also investigated for chikungunya and dengue viruses.

## Methods

We studied patients who had developed a new neurological condition associated with suspected Zika virus infection, whose samples had been submitted to the Flavivirus Reference Laboratory of the Instituto Oswaldo Cruz (Fiocruz), Rio de Janeiro.

### Ethics statement

The study protocol was approved by the Comitê de Ética em Pesquisa do Instituto Nacional de Infectologia Evandro Chagas (reference 59254116.0.1001.5262). Patient-identifying data was anonymised.

### Study population

**Fig 1:**
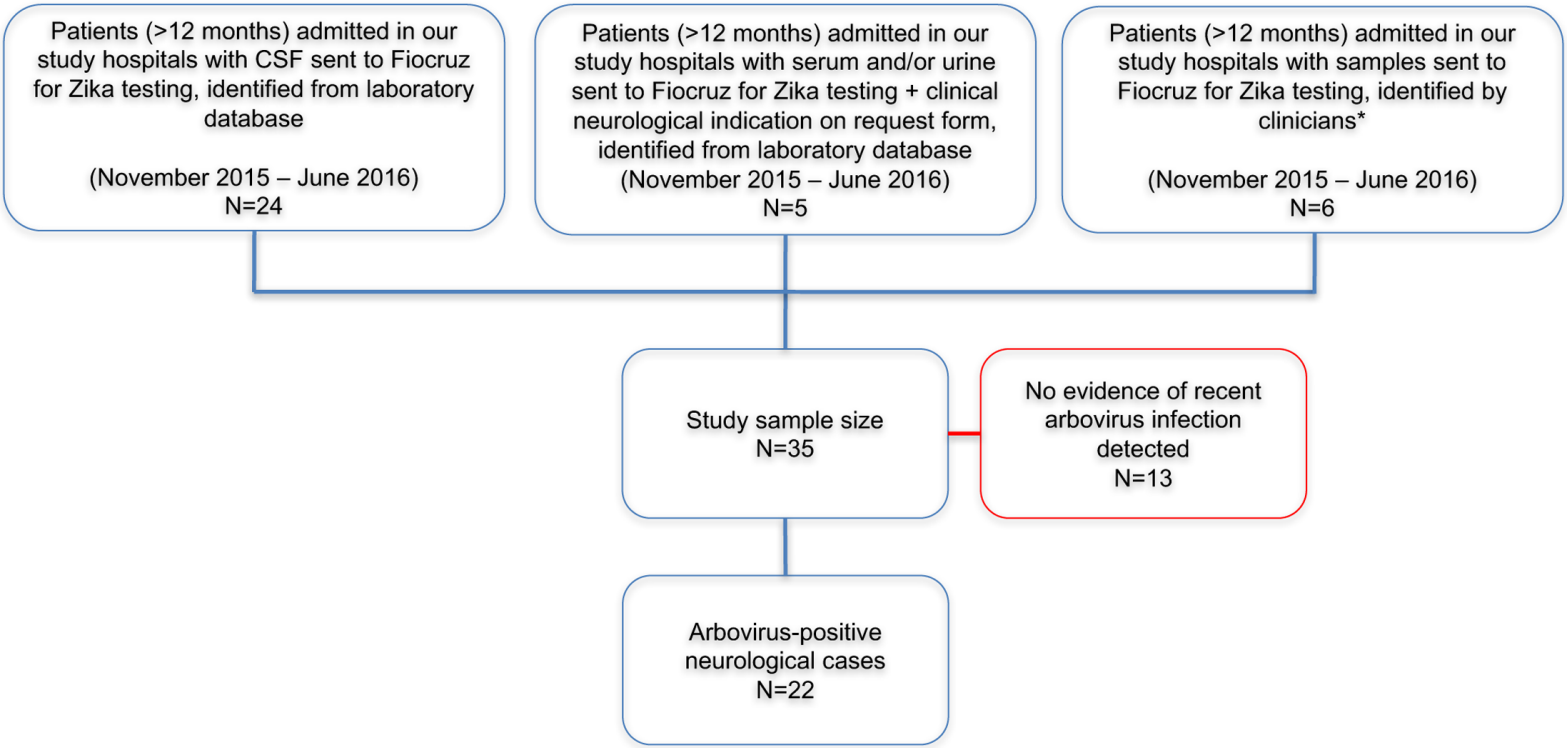
Study population of patients with neurological disease associated with suspected Zika virus infection. *These patients did not appear in the laboratory database search because their CSF sample was not recorded on the database and no clinical information was included in request forms for serum and/or urine. They were identified by the clinicians who had previously managed their care in our study hospitals.

We studied patients admitted in 11 hospitals (appendix) in Rio de Janeiro from 1^st^ November 2015 to 1^st^ June 2016, who had presented with an acute neurological condition associated with a suspected Zika virus infection, as identified by fever, arthralgia or rash illness in the preceding three months. In this evolving epidemic situation we used three approaches to identify patients: using the laboratory database, we retrospectively identified 29 patients who had had either cerebrospinal fluid (CSF) regardless of indication, or serum and/or urine in the context of neurological disease, sent to the laboratory for Zika virus diagnostics; additionally, clinicians from our study hospitals identified six further patients, whose CSF sample was not on the database and whose serum and/or urine request forms did not have an indication (Fig 1). Patients under the age of 12 months were excluded.

Clinical information was obtained from case notes and discussion with the patients’ clinicians and documented on standardised case report forms by a member of the study team. The information obtained included demographics, past medical history, admission history, examination, investigations, diagnosis, and management. Investigations included brain and spine imaging for patients with suspected central nervous system infection, and nerve conduction studies with or without electromyography for those with peripheral disease. Nerve conduction study results were reviewed by an independent expert neurophysiologist to ensure consistency. The Brighton criteria were used to indicate the level of certainty for diagnosing GBS and similar criteria were applied for radiculitis, encephalitis, myelitis, and meningitis (appendix). We determined whether patients had peripheral nervous system disease (GBS, radiculitis), CNS disease (encephalitis, myelitis, meningitis) or both.

### Laboratory testing

CSF, serum, and urine samples were tested for evidence of Zika, chikungunya, and dengue virus infection at the Fiocruz Flavivirus Laboratory. We considered detection of viral RNA and/or IgM-specific antibody in the CSF as evidence of recent CNS infection as previously;[22] IgM antibody in the serum, or RNA in the serum or urine was taken as evidence of systemic infection.

An expanded protocol based on the interim recommendations from the WHO for laboratory testing for Zika virus was followed:[23] RNA was extracted from 140μl of CSF, serum, and urine samples and eluted in 50μl using the Qiamp Mini Elute Virus Spin Kit from Qiagen (Brazil). The CSF, serum, and urine samples were tested by qRT-PCR for detection of Zika, chikungunya, and dengue virus RNA as described previously.[24] Serum IgM and IgG antibodies to Zika virus NS1 antigen and serum and CSF IgM and IgG antibodies to chikungunya virus were measured using commercial ELISAs (Euroimmun, Luebeck, Germany), according to the manufacturer’s protocol.[25–27] CSF IgM antibodies to Zika virus were measured using a recommended capture ELISA based on the US Centers for Disease Control and Prevention (CDC) emergency use authorization protocol (CDC Fort Collins, CO, USA).[28] Serum and CSF IgM and IgG antibodies to dengue virus were measured using commercial ELISAs (Panbio, Brazil). For serum samples with sufficient volume remaining, anti-ganglioside antibodies, which are associated with GBS and other autoimmune neuropathies, were tested by ELISA (Bühlmann-Gangliocombi, Schönenbuch, Switzerland) following the manufacturer's instructions.

### Statistical analysis

The median time from illness onset to the development of neurological symptoms was compared for those with CNS and peripheral nervous system disease, and for those with or without CNS Zika virus infection, using the Wilcoxon-Mann-Whitney U-test.

## Results

**Fig 2:**
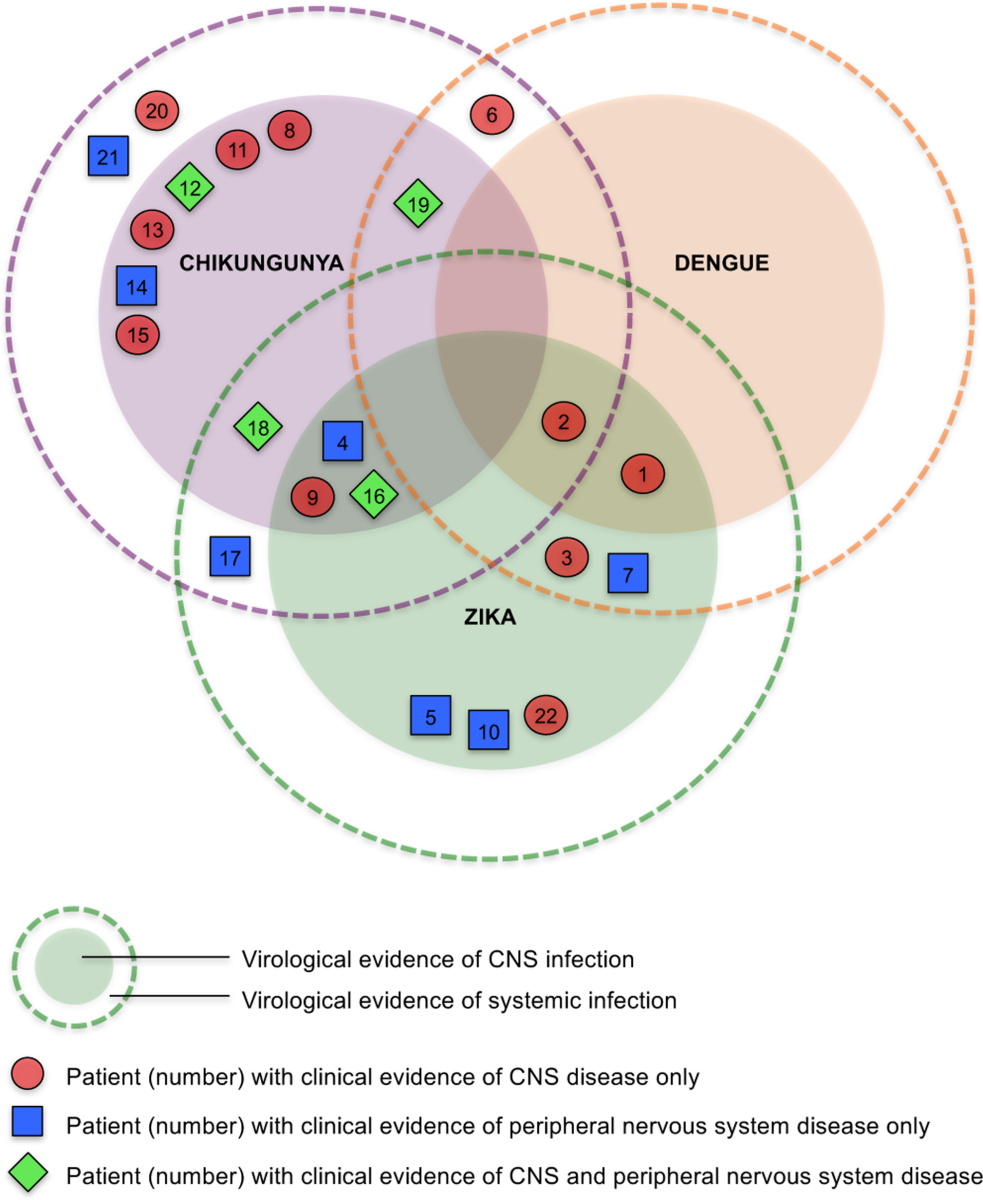
Venn diagram for 22 patients showing virological evidence of CNS or systemic infection with Zika, chikungunya and/or dengue, and clinical presentation with CNS or peripheral nervous system disease. We distinguish *virological* evidence of CNS or systemic *infection* (based on PCR/antibody testing) from *clinical* evidence of CNS or peripheral nervous system *disease* (based on clinical features). Patients in the inner darker circles have evidence of CNS +/- systemic infection with the respective virus. Those in the outer paler circles have evidence of only systemic infection with the respective virus. Note that patients 1 and 3 had confirmed Zika, +/- dengue; patients 2 and 7 had Zika or dengue or both.

We identified 35 patients with new neurological disease associated with a suspected Zika virus infection. Evidence for recent arbovirus infection was found in 22 (63%) of them. Table 1 and Fig 2 show the virological diagnosis for each patient, taking into account any potential serological cross-reactivity between flaviviruses.

**Table 1:** Virological evidence for Zika, chikungunya and/or dengue virus infection in 22 patients presenting with acute neurological disease, ordered by date of admission.

Patient	Zika					Chikungunya					Dengue					Other CSF investigations	Virological Diagnosis
	CSF PCR	CSF IgM	Serum PCR	Serum IgM	Urine PCR	CSF PCR	CSF IgM	Serum PCR	Serum IgM	Urine PCR	CSF PCR	CSF IgM	Serum PCR	Serum IgM	Urine PCR		
1*	-	+	-	-	+	-	-	-	-	-	-	+	-	+	-	Neg: MCS, HSV, VDRL, CRAG	Zika-CNS +/- Dengue-CNS †
2	-	+	-	-	-	-	-	-	+	-	-	+	-	+	-	na	Zika-CNS or Dengue-CNS or both, Chik-Syst
3	-	+	+	-	+	-	-	-	-	-	-	-	-	+	-	Neg: HSV	Zika-CNS +/- Dengue-Syst †
4	+	+	-	-	na	+	-	+	-	na	-	-	-	-	na	Neg: MCS	Zika/Chik-CNS
5	-	+	-	-	+	-	-	-	-	-	-	-	-	-	-	Neg: MCS, VDRL	Zika-CNS
6	na	na	-	-	-	na	na	-	+	-	na	na	-	+	-	Neg: MCS, VDRL	Chik/Dengue-Syst
7	-	+	-	+	-	-	-	-	-	-	-	-	-	+	-	na	Zika-CNS or Dengue-Syst or both
8	-	-	-	-	-	+	-	-	-	-	-	-	-	-	-	Neg: MCS	Chik-CNS
9	-	+	na	na	na	+	+	na	na	na	-	-	na	na	na	Neg: HSV	Zika/Chik-CNS
10	-	+	-	+	+	-	-	-	-	-	-	na	-	-	-	Neg: MCS	Zika-CNS
11	-	-	-	-	-	+	+	-	+	+	-	-	-	-	-	Neg: MCS, VDRL	Chik-CNS
12	-	-	na	na	-	+	+	na	na	-	-	-	na	na	-	na	Chik-CNS
13	-	-	-	-	-	+	-	+	+	-	-	-	-	-	-	Neg: MCS, VDRL, CRAG, CMV/VZV/HSV	Chik-CNS
14	-	-	na	na	na	+	-	na	na	na	-	-	na	na	na	na	Chik-CNS
15	-	-	na	na	na	+	-	na	na	na	-	-	na	na	na	Neg: HSV	Chik-CNS
16	+	+	na	na	-	+	+	na	na	-	-	-	na	na	-	na	Zika/Chik-CNS
17	na	na	-	-	+	na	na	+	-	-	na	na	-	-	-	Pos: VDRL	Zika/Chik-Syst
18	-	-	na	na	+	+	+	na	na	-	-	-	na	na	-	Neg: HSV	Chik-CNS, Zika-Syst
19	-	-	-	-	-	+	+	+	+	-	-	-	-	+	-	na	Chik-CNS, Dengue-Syst
20	-	-	-	-	-	-	-	+	+	+	-	-	-	-	-	na	Chik-Syst
21	-	-	-	-	-	-	-	-	+	+	-	-	-	-	-	Neg: MCS, VDRL	Chik-Syst
22	-	+	-	-	-	-	-	-	-	-	-	-	-	-	-	na	Zika-CNS

"+" = positive, "-" = negative, "na" = sample not available or inadequate volume; PCR = polymerase chain reaction; CSF = cerebrospinal fluid; MCS = microscopy, culture and sensitivity; HSV = herpes simplex virus, CMV = cytomegalovirus, VZV = varicella zoster virus, VDRL = venereal disease research laboratory (syphilis), CRAG = cryptococcal antigen, Chik = chikungunya; CNS = virus detected in central nervous system, Syst = virus detected systemically (i.e. outside CNS) only. Zika virus PCR primers used: 1086–1102, 1107–1137.[24] See appendix for antibody levels, IgG results and time between infection and sample collection.

*Preliminary information for this patient has previously been published.

†These patients had PCR evidence of Zika virus infection, with serological evidence of dengue infection potentially secondary to cross-reactivity.

Twelve (34%) had evidence of Zika virus infection. Three (9%) with evidence of Zika virus infection alone presented with GBS (two patients) or encephalitis (one). Nine (26%) with evidence of Zika also had evidence for another arbovirus; five with chikungunya, three with dengue, one with both chikungunya and dengue. Three of them presented with GBS, including one with the facial diplegia and paraesthesia variant, and six had CNS infection, predominantly encephalitis and/or myelitis. Ten patients (29%) negative for Zika had evidence for another arboviral infection; eight with chikungunya and two with chikungunya and dengue. Two of these presented with GBS and eight with CNS disease.

The remaining 13 patients (37%), with no evidence of a recent Zika, chikungunya, or dengue virus infection, presented with GBS (six patients) or CNS disease (seven). Their diagnoses are given in the appendix, but they are not considered further here.

### Clinical features

All 22 patients with evidence of recent arbovirus infection were Brazilian nationals with no recent travel history, from 18 different districts of Rio de Janeiro. The median (range) age was 51·5 (17–84) years. The male:female ratio was 1:1. Their individual clinical features are summarised in Table 2 and a detailed example of a clinical case, patient 9, is described in Box 1.

**Table 2:** Individual clinical features of 22 patients presenting with neurological disease associated with Zika, chikungunya and/or dengue virus infection, ordered by date of admission.

Patient	Virological Diagnosis	Systemic Features	Prodrome Length (days)	Neurological Features	CSF WCC	CSF Protein	Anti-ganglioside Antibody	Neurological Diagnosis (levels of certainty)	Management	Progress and Outcome
1 (F/47)	Zika-CNS +/- Dengue-CNS	Rash, arthralgia, malaise	4	Confusion, dysarthria, drowsiness; paraparesis; GCS 3; CT head—diffuse white matter hypodensities	10	1·11	GM1	Encephalitis (I)	Mannitol	ICU; intubated; patient rapidly deteriorated and died
		WCC 8·0								
2 (F/59)	Zika-CNS or Dengue-CNS or both, Chik-Syst	Fever, rash, arthralgia	7	UL and LL paraesthesia; spastic quadraparesis; extensor plantars; impaired UL and LL LT, PP, Vi, Pr; urinary retention; GCS 15; MRI spine—intramedullary signal abnormality involving cervical and thoracic cord; MRI brain—normal; NP—normal	4	0·36	GM1, GD1a, GD1b	Myelitis (I)	IVIG; steroids x 2	Developed pulmonary oedema on IVIG; responded to steroids, mRS 3 at 4 months
		WCC 8·1								
3 (M/26)	Zika-CNS +/- Dengue-Syst	Fever	1	Confusion; truncal, UL and LL paraesthesia and numbness; spastic hyperreflexic quadraparesis; T5 sensory level; LMN facial nerve and supranuclear gaze palsies; impaired UL and LL LT, PP, Vi, Pr; urinary incontinence; GCS 13; MRI brain and spine—signal abnormality involving cerebellar peduncles, medulla and intramedullary cervical cord	100	1·12	GM1, GD1a, GD1b	Encephalo-myelitis (I,I)	IVIG; steroids	ICU; intubated; improved, mRS 1 at 4 months
4 (M/34)	Zika/Chik-CNS	Rash	12	UL and LL paraesthesia; mild ataxia; bilateral LMN facial nerve palsy; hyperreflexic LL; GCS 15; MRI brain—gadolinium enhancement of bilateral facial nerves; NP—normal	2	0·69	-	GBS variant (facial diplegia with paraesthesia)	IVIG	Improved, full recovery at 2 months
5 (F/41)	Zika-CNS	Fever, rash, malaise	5	LL and peri-orbital paraesthesia; normotonic areflexic paraparesis; impaired LL LT, PP, Vi; GCS 15; CT brain—normal	0	2·07	na	GBS (II)	IVIG	Improved (extent unknown)
		WCC 21·8								
6 (F/30)	Chik/Dengue-Syst	Fever, rash, malaise	27	LL paraesthesia; normoreflexic paraparesis; urinary retention; GCS 15; MRI brain and spine—normal; NP—normal	0	0·21	-	Myelitis (II)	IVIG; steroids	Improved (extent unknown)
		WCC 9·3								
7 (F/66)	Zika-CNS or Dengue-Syst or both	Fever, arthralgia, malaise	13	UL and LL paraesthesia; flaccid areflexic quadraparesis; bilateral LMN facial nerve palsy; impaired UL and LL LT, PP, Vi; GCS 15; NP—AMSAN	0	0·95	GD1a	GBS (I)	IVIG	ICU; intubated; improved at 1 week (extent unknown)
		WCC 7·7								
8 (M/20)	Chik-CNS	Fever	0	R UL and LL paraesthesia and triparesis (L UL spared), hyperreflexic LL; impaired LL LT, PP, Vi, Pr; C6 sensory level; urinary retention; GCS 15; MRI spine—signal abnormality involving cervical and thoracic cord; MRI brain normal; NP—normal	0	0·29	-	Myelitis (I)	Steroids x 2	Improved, mRS 2 at 3 weeks
		WCC 15·4								
9 (F/80)	Zika/Chik-CNS	Fever, rash, arthralgia, malaise	5	Headache, confusion; flaccid hyporeflexic quadraparesis; GCS 14; MRI brain and spine—signal abnormality involving anterior medulla, anterior cervical and thoracic cord, temporal lobes, amygdala, small area adjacent to temporal horn of L lateral ventricle, pachymeningeal enhancement	117	1·74	na	Encephalo-myelitis (I,I) with subclinical meningitis	IVIG	ICU; developed sacral osteomyelitis; improved, mRS 4 at 2 months
		PLT 134								
		WCC 7·5								
10 (M/38)	Zika-CNS	Fever, rash, malaise	10	LL paraesthesia; flaccid areflexic quadraparesis; L LMN facial nerve palsy; impaired LL Pr; GCS 15	1	1·72	-	GBS (II)	IVIG; antivirals	ICU; intubated, ventilator-associated pneumonia; improved (extent unknown)
		PLT 220								
		WCC 14·0								
11 (M/76)	Chik-CNS	Rash, arthralgia	0	2 seizures; confusion, dysarthria, headache, neck stiffness; spastic paraparesis; extensor plantars, palmomental reflex; LL neuropathic pain; T2-3 sensory level; urinary incontinence; GCS 14	80	1·45	GD1a	Meningo-encephalo-myelitis (III,I,III)	Antivirals; antibiotics	Unknown outcome
		PLT 254								
		WCC 13·0								
12 (M/63)	Chik-CNS	Fever, rash, arthralgia, malaise	2	LL paraesthesia; flaccid areflexic paraperesis; T4 sensory level; urinary retention; fell with intracranial injury; GCS 15 on admission, 12 after fall; CT brain normal, MRI brain and spine normal; NP—AMSAN	0	0·92	-	Myeloradiculitis (II)	IVIG	ICU, intubated (after fall and head injury); no improvement; mRS 5 at 2 months
		PLT 116								
		WCC 6·5								
13 (F/51)	Chik-CNS	Fever, arthralgia, malaise	6	Confusion, 1 x seizure, drowsiness, dysarthria; GCS 3; CT head normal	11	0·45	GQ1b	Encephalitis (I)	Antivirals	Improved, full recovery at 2 months
14 (M/45)	Chik-CNS	Fever, arthralgia, malaise, diarrhoea	29	UL and LL paraesthesia; flaccid areflexic quadraparesis; impaired UL and LL LT, PP; GCS 15	0	0·75	na	GBS (II)	IVIG	ICU; improved mRS 3
		PLT 203								
		WCC 8·7								
15 (M/84)	Chik-CNS	Fever, rash, arthralgia, malaise, diarrhoea	4	Confusion, impaired speech and swallow; flaccid hyporeflexic quadraparesis; myalgia; GCS 8; MRI brain—focal areas of hyperintensity likely related to microangiopathy; NP—inflammatory myopathy	42	1·11	na	Encephalitis (I), Myositis	IVIG; antivirals; antibiotics; antifungals	Ventilator-associated pneumonia; no improvement, mRS 5 at 6 weeks
		PLT 80								
		WCC 16·3								
16 (M/65)	Zika/Chik-CNS	Fever, rash, arthralgia, malaise	0	LL paraesthesia; flaccid areflexic paraparesis; T11 sensory level; impaired LL sensation LT, PP, Vi, Pr; urinary retention; GCS 15; MRI brain and spine normal; NP—AMSAN	10	1·08	na	Myeloradiculitis (I)	IVIG; steroids	No improvement at 3 weeks
		PLT 130								
		WCC 9·9								
17 (F/19)	Zika/Chik-Syst	Rash	41	UL and LL paraesthesia; quadraparesis, hyporeflexic LL; GCS 15; CT brain normal; NP—AMAN; patient 19 weeks pregnant at admission, foetus diagnosed with Dandy-Walker syndrome	2	0·23	-	GBS (II)	IVIG	No improvement at 3 weeks
		PLT 537								
		WCC 9·0								
18 (F/56)	Chik-CNS, Zika-Syst	Fever, rash, malaise	4	LL paraesthesia; flaccid areflexic quadraparesis; impaired UL & LL LT, PP, Vi, Pr; C7 sensory level; urinary retention; GCS 15; MRI brain and spine normal; NP—AMSAN	0	0·71	na	Myeloradiculitis (II)	IVIG; steroids	No improvement, mRS 5 at 1 month
		PLT 340								
		WCC 15·0								
19 (M/62)	Chik-CNS, Dengue-Syst	Fever, rash	8	LL paraesthesia; normotonic hyperreflexic paraplegia; extensor plantars; impaired LL Vi; T6-8 sensory level; urinary incontinence; GCS 15; CT brain normal, MRI brain and spine normal; NP—AIDP	16	1·09	-	Myeloradiculitis (I)	IVIG; steroids	Improved, mRS 2 at 1 month
		PLT 680								
		WCC 18·8								
20 (M/17)	Chik-Syst	Fever, rash	16	L sided numbness (LL, UL, truncal); L hemiparesis; L UMN facial nerve palsy; headache; impaired L sided UL & LL LT, PP; GCS 15; MRI brain—demyelinating lesions R parietal lobe & thalamus consistent with ADEM	na	0·44	na	ADEM	IVIG; steroids	Improved, mRS 2 at 3 weeks
		PLT 362								
		WCC 6·3								
21 (F/67)	Chik-Syst	Fever, arthralgia	7	Flaccid areflexic quadraparesis; dysphagia; palatal weakness; dyspnoea; impaired UL & LL Pr, LL Vi; GCS 15; CT brain normal; NP—AMSAN	10	0·71	-	GBS (I)	IVIG x 2	ICU; intubated; no improvement, mRS 5 at 2 weeks
		PLT 397								
		WCC 8·9								
22 (F/52)	Zika-CNS	Fever	0	LL and R facial numbness, impaired co-ordination; headache, diplopia; GCS 15; MRI brain—signal abnormality involving frontal and parietal lobes, pons and right cerebellar peduncle	6	0·37	GD1a	Encephalitis*	Steroids	Improved, mRS 0 at 2 months
		PLT 412								
		WCC 10·2								

Prodrome length = interval between onset of infection and neurological illness; Chik = chikungunya; CNS = virus detected in central nervous system, Syst = virus detected systemically (i.e. outside CNS) only; PLT = platelet count x10^9^/L, WCC = white cell count (systemic x10^9^/L, CSF /μL); L = left, R = right, LL = lower limb, UL = upper limb, CSF = cerebrospinal fluid, LMN = lower motor neuron, UMN = upper motor neuron, LT = light touch, PP = pinprick, Vi = vibration, Pr = proprioception, GCS = Glasgow coma scale; MRI = magnetic resonance imaging, CT = computed tomography, NP = neurophysiology; AMSAN = acute motor and sensory axonal neuropathy; AMAN = acute motor axonal neuropathy; AIDP = acute inflammatory demyelinating polyneuropathy; GBS = Guillain-Barré syndrome, ADEM = acute disseminated encephalomyelitis; IVIG = intravenous immunoglobulin; ICU = intensive care unit admission, mRS = modified Rankin Scale; "na" = not available. For neurological diagnoses the levels of diagnostic certainty are indicated I-III (highest to lowest), as per the Brighton and other criteria (appendix). The time post-onset of neurological symptoms is given for outcomes, where known.

*Although the GCS score was 15, the clinical features and imaging indicated focal encephalitis.

**Box 1 Clinical presentation of encephalomyelitis (with subclinical meningitis) associated with Zika and chikungunya virus infections (patient 9)**

An 80-year-old Brazilian female with a past medical history of hypertension, obesity and osteoarthritis presented with a one-day history of headache, confusion, and symmetrical weakness in all four limbs. Five days earlier, she had fever, rash, arthralgia and general malaise. There was no preceding history of respiratory or gastrointestinal infection, or recent vaccination, and she did not have any bowel or bladder symptoms.

On examination, she had a diffuse, centrifugal, erythematous rash, with mild joint swelling of the knees. She was mildly confused with a Glasgow Coma Scale of 14. She had a flaccid tetraparesis, worse proximally (Medical Research Council grade 1 in all four limbs) than distally (Medical Research Council grade 3 in all four limbs). Her reflexes were present but depressed throughout. As far as assessment would allow, light touch, vibration, temperature and proprioception appeared normal. There was no sensory level apparent, or cranial nerve involvement.

**Fig 3:**
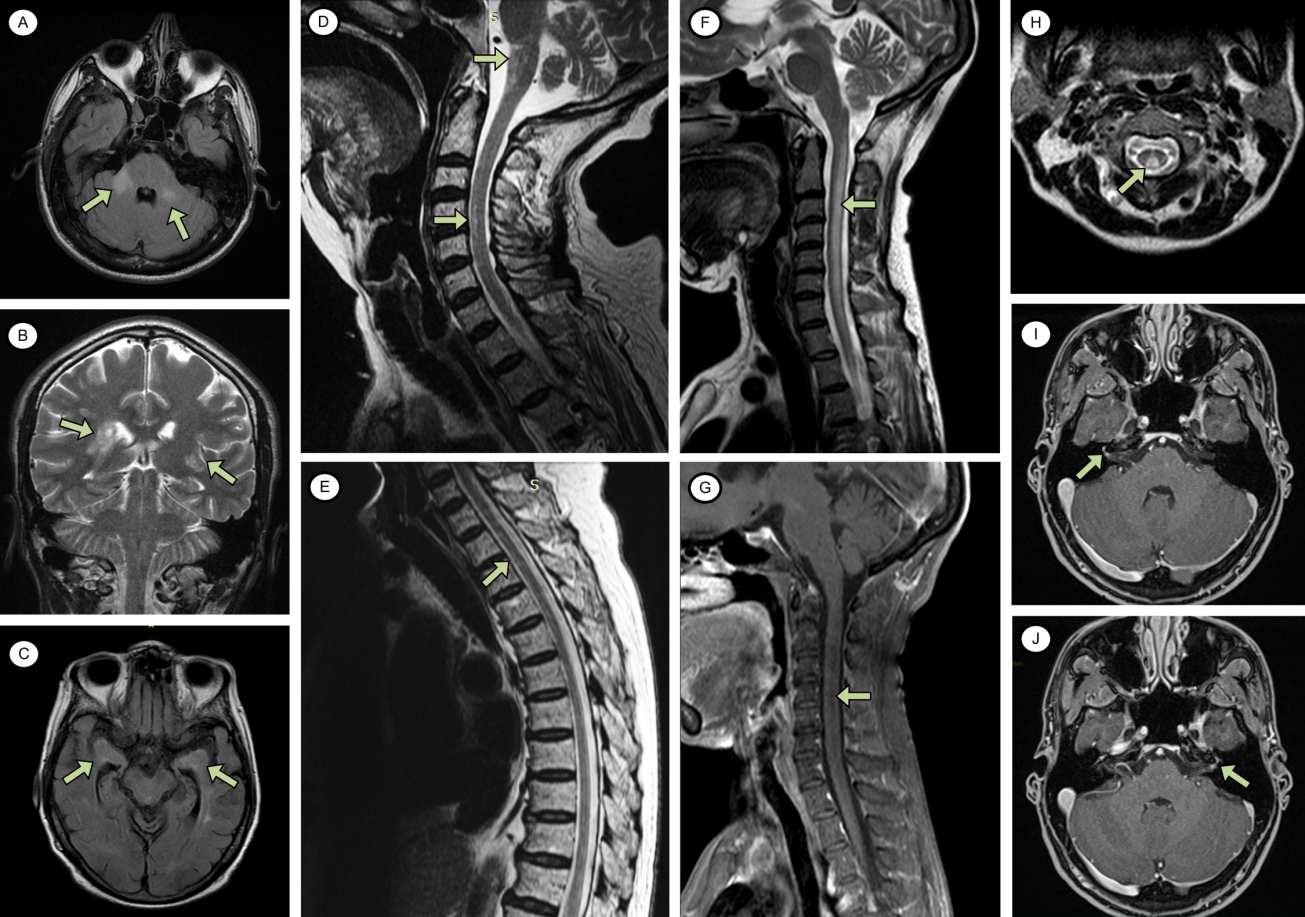
Central nervous system (CNS) imaging abnormalities in patients with evidence of Zika, chikungunya and/or dengue virus infection. A: Encephalomyelitis in a patient with CNS Zika and systemic dengue infection (patient 3). Fluid attenuation inversion recovery [FLAIR] signal abnormality involving the middle cerebellar peduncles, more marked on the right (axial scan). B: Acute disseminated encephalomyelitis in a patient with systemic chikungunya infection (patient 20). Confluent areas of T2 signal abnormality suggesting neuroinflammation consistent with demyelination (coronal scan). C, D, E: Encephalomyelitis with subclinical meningitis in a patient with CNS Zika + chikungunya infection (patient 9). FLAIR signal abnormality involving the medial temporal lobes, amygdala, and a small area of abnormality adjacent to the temporal horn of the left lateral ventricle (C, axial scan). High signal intensity on T2-weighted images in the anterior medulla, and anterior cervical and thoracic cord (D and E, sagittal scans). F, G, H: Myelitis in a patient with CNS Zika + dengue and systemic chikungunya infection (patient 2). Extensive intramedullary signal abnormality of the cervical cord, without evidence of contrast enhancement (F, sagittal T2-weighted scan; G, sagittal T1-weighted scan with gadolinium; H, axial T2-weighted scan). I, J: Facial diplegia with paraesthesia in patient with CNS Zika + chikungunya infection (patient 4). Bilateral facial nerve enhancement on T2-weighted images with gadolinium (axial scan).

A lumbar puncture revealed a predominantly lymphocytic CSF pleocytosis of 117 leucocytes/μl, elevated protein of 1.74 g/dL and glucose of 3.9 mmol/L. PCR for HSV 1 and 2 was negative. She had a mild thrombocytopenia of 134 x 10^9^/L and raised creatinine of 160 µmol/L. An MRI scan of her brain and spine showed signal abnormality involving the anterior medulla, anterior cervical and thoracic cord, temporal lobes, amygdala and a small area adjacent to temporal horn of her left lateral ventricle, with pachymeningeal enhancement (Fig 3C, 3D and 3E). On the basis of the clinical and imaging findings she was diagnosed with encephalomyelitis (with subclinical meningitis) and admitted to the intensive care unit, where her upper limb power marginally improved without treatment. A course of intravenous immunoglobulin therapy did not have any further effect. The patient was discharged to a care home and despite physiotherapy was still unable to walk after seven months.

Her CSF was positive by IgM ELISAs for Zika and chikungunya viruses. PCR was also positive for chikungunya, but not Zika. There was with no evidence of dengue virus infection.

The initial symptoms of arboviral infection included fever (82% of the patients), rash (68%), malaise (55%) and arthralgia (50%). Two patients had diarrhoea in the month preceding their neurological illness (Table 2); no patient reported a preceding lower respiratory tract infection or conjunctivitis. Five (patients 11, 13, 15, 16, 22) reported previous dengue. One (patient 22) reported prior vaccination against yellow fever. The median (range) time between infective symptoms and onset of neurological disease was 6·5 (0–41) days. Patients presented with a range of neurological syndromes affecting the CNS, peripheral nervous system, or both, as detailed in Table 2.

The seven patients with GBS all had a preceding febrile and/or rash syndrome, which was a median (range) 12 (5–41) days before the neurological presentation; there was no statistically significant difference in prodrome length between those with and without CNS Zika infection. The presentations for six patients were similar—typically paraesthesia (five patients) with a rapidly ascending symmetrical flaccid paralysis, involving all four limbs in five patients, or the legs only in one (patient 5). Another (patient 4) presented with a GBS variant, with bilateral lower motor neuron facial nerve palsies and paraesthesia in all four limbs.

Fifteen patients presented with CNS disease, including encephalitis and/or myelitis, with or without involvement of the meninges or peripheral nerves. All had a febrile or rash syndrome, many with arthralgia and malaise. The median (range) time delay between this systemic illness and neurological disease was 4 (0–27) days; for these patients with CNS disease, there was no statistically significant difference in prodrome length between those with and without CNS Zika infection.

The eight patients with encephalitis (with or without other CNS disease) had a median (range) Glasgow Coma Scale score of 13·5 (3–15); six were confused, and two had seizures. One patient had a supranuclear gaze palsy; two had facial weakness and four had difficulties with speech or swallowing. The ten patients with myelitis (with or without encephalitis) comprised five with paraparesis (one spastic, two flaccid, and two with normal tone), four with quadraparesis (two spastic, two flaccid) and one with a triparesis. Seven of these patients had a sensory level, six had urinary retention and three had urinary incontinence. Three patients with flaccid paresis had signs and symptoms compatible with transverse myelitis, namely a sensory level and urinary retention. However, one patient with encephalomyelitis with flaccid areflexic quadraparesis (patient 9) had extensive imaging changes in the anterior of the cord consistent with poliomyelitis-like anterior horn cell damage.

Neurophysiological studies (see below) confirmed the involvement of lower motor neurons for four patients with myelitis: two of those with flaccid paraparesis, one with flaccid quadraparesis, and one with paraparesis and normal tone. The clinical characteristics of the patients with central, peripheral, and mixed nervous system disease are compared in Table 3.

**Table 3:** Clinical characteristics of 22 patients presenting with neurological disease associated with Zika, chikungunya and/or dengue virus infection.

	n (%) or median (IQR)			
	All patients (n = 22)	CNS disease (n = 11)	PNS disease (n = 7)	CNS & PNS disease (n = 4)
Age (years)	51·5 (35–64·5)	51 (28–67·5)	41 (35–55·5)	62·5 (60·5–63·5)
Males	11 (50%)	5 (45%)	3 (43%)	3 (75%)
Previous yellow fever vaccination (of 12 patients)	1 (8%)	1 of 4 (25%)	0 of 4 (0%)	0 (0%)
Previous dengue (of 20 patients)	5 (25%)	4 (36%)	0 of 5 (0%)	1 of 3 (33%)
Co-morbidity (of 20 patients)	10 (50%)	5 (45%)	2 of 6 (33%)	3 of 3 (100%)
* *Diabetes mellitus (type II)	2 (10%)	2 (18%)	0 of 6 (0%)	0 of 3 (0%)
* *Stroke	2 (10%)	0 (0%)	2 of 6 (33%)	0 of 3 (0%)
* *Hypertension	6 (30%)	3 (27%)	0 of 6 (0%)	3 of 3 (100%)
* *Hypercholesterolaemia	2 (10%)	1 (9%)	1 of 6 (17%)	0 of 3 (0%)
* *Cancer	2 (10%)	1 (9%)	0 of 6 (0%)	1 of 3 (33%)
* *Asthma	1 (5%)	1 (9%)	0 of 6 (0%)	0 of 3 (0%)
* *Cardiac disease	1 (5%)	0 (0%)	1 of 6 (17%)	0 of 3 (0%)
* *Neurological disease (Tourette’s syndrome)	1 (5%)	1 (9%)	0 of 6 (0%)	0 of 3 (0%)
Systemic features				
* *Fever	18 (82%)	9 (82%)	5 (71%)	4 (100%)
* *Rash	15 (68%)	7 (64%)	4 (57%)	4 (100%)
* *Arthralgia	11 (50%)	6 (55%)	3 (43%)	2 (50%)
* *Malaise	12 (55%)	5 (45%)	4 (57%)	3 (75%)
* *Diarrhoea	2 (9%)	1 (9%)	1 (14%)	0 (0%)
Prodrome length (days)	5·5 (2·5–11·5)	4 (0·5–6·5)	12 (8·5–21)	3 (1·5–5)
Neurological symptoms				
* *Weakness	19 (86%)	9 (82%)	6 (86%)	4 (100%)
* *Sensory disturbance	17 (77%)	7 (64%)	6 (86%)	4 (100%)
Neurological examination (of 21 patients)				
* *Cranial nerve involvement	6 (29%)	2 of 10 (20%)	4 (57%)	0 (0%)
* *Sensory level	8 (38%)	4 of 10 (40%)	0 (0%)	4 (100%)
* *GCS<15 on admission	8 (38%)	6 of 10 (60%)	1 (14%)	1 (25%)
* *Diminished or absent reflexes	11 (52%)	2 of 10 (20%)	6 (86%)	3 (75%)
* *Brisk reflexes	6 (29%)	4 of 10 (40%)	1 (14%)	1 (25%)
Lumbar puncture results				
* *CSF white cell count (of 21 patients)	4 (0–11)	10·5 (4·5–70·5)	1 (0–2)	5 (0–11·5)
* *CSF protein	83·5 (44·25–111)	45 (36·4–111·5)	75 (70–133.2)	100 (86·8–108·3)
Treatment				
* *IVIG	17 (77%)	6 (55%)	7 (100%)	4 (100%)
* *Steroids	9 (41%)	6 (55%)	0 (0%)	3 (75%)
Outcome				
* *Responded to treatment (of 21 patients)	14 (67%)	7 of 10 (70%)	5 (71%)	2 (50%)
* *Admitted to ICU	8 (36%)	3 (27%)	4 (57%)	1 (25%)
* *Intubated	6 of 21 (29%)	2 of 10 (20%)	3 (43%)	1 (25%)
* *Died	1 (5%)	1 (9%)	0 (0%)	0 (0%)

CNS = central nervous system; PNS = peripheral nervous system. For certain parameters we did not have data for all patients; the number of patients for whom data was available is indicated in brackets.

### Virology and serology

Zika virus RNA was detected in two CSF, one serum and six urine samples (from eight patients); Zika IgM was detected in 10 CSF and two serum samples (10 patients). Chikungunya virus RNA was detected in 11 CSF, six serum, and three urine samples (14 patients); chikungunya IgM was detected in six CSF and seven serum samples (11 patients). Dengue virus RNA was not found; dengue IgM was found in two CSF and six serum samples (six patients).

As expected, many patients had serum IgG against dengue virus, consistent with prior exposure (appendix). Of the 13 patients with no evidence of recent arbovirus infection, serum Zika virus IgG was detected in four. Anti-GM1, GD1a, GD1b and GQ1b antibodies were found in the serum of patients with both peripheral and central nervous system disease (Table 2).

### Investigations

Ten out of the 22 patients had a CSF pleocytosis (≥5/μL, all showing predominantly lymphocytes/monocytes) and 15 had a CSF protein above 0·45 g/L. Four patients had a mild thrombocytopenia and seven had a peripheral leucocytosis, but none had leucopenia. Imaging studies are detailed in Table 2, with examples shown in Fig 3. High signal changes were found in the cervical (four patients) and thoracic (two) cord, brainstem (two), cerebellar peduncles (two) and cortex (three); demyelination of the parietal cortex and thalamus was reported in patient 20 and pachymeningeal enhancement in patient 9. Three different neurophysiological patterns were seen in patients with peripheral involvement—acute motor axonal neuropathy (AMAN), acute motor and sensory axonal neuropathy (AMSAN) and acute inflammatory demyelinating polyneuropathy (AIDP) (see appendix for original data). AMAN and AMSAN were seen in patients both with and without probable Zika virus infection; AIDP was seen only in one patient with CNS chikungunya virus infection.

### Outcome

Eight (36%; four with confirmed Zika) of the 22 patients required admission to an intensive care unit; six (27%) needed intubation (including half the patients with GBS). Ten patients were treated with intravenous immunoglobulin, two with corticosteroids, and seven with both; four with encephalitis received aciclovir as presumptive treatment for herpes simplex virus encephalitis. Four patients developed hospital acquired infections, including ventilator-associated pneumonia and sacral osteomyelitis secondary to immobility. Fourteen (of 21 with outcome data, 67%; six with confirmed Zika) patients improved; one with evidence of Zika +/- dengue infection of the CNS deteriorated rapidly and died.

## Discussion

With 84 countries or territories now affected by Zika virus,[29] increasing reports of associated neurological disease other than GBS,[10–13, 15, 16] and growing concern about coinfections with other arboviruses,[30] there is an urgent need to determine the full spectrum of Zika’s neurological complications and its relationship with other arboviruses. In our study, which begins to address these questions, over half of the patients with Zika virus infection had presentations other than GBS, suggesting that these complications may be more important than recognised previously. Patients had involvement of the meninges, brain parenchyma, spinal cord, and peripheral nerves in various combinations, as evidenced by the clinical features, imaging and neurophysiological findings, and as has been described for other flaviviruses.[9]

Four patients with evidence of Zika virus infection also had CNS infection with chikungunya virus. Ten further patients negative for Zika tested positive for chikungunya. In South America, reports of neurological disease associated with chikungunya virus are scarce, which may reflect a lack of awareness among clinicians about the potential to affect the nervous system, or the relatively recent arrival of the virus. Interestingly, in one patient (patient 17) virus was detected 30 days after the onset of neurological disease, suggesting a persistent infection or a late coincidental infection. In our study of patients from Rio de Janeiro, chikungunya virus was as important a cause of neurological disease as Zika virus. Its importance in other settings where Zika virus is assumed to be the cause of febrile illness, with or without neurological disease, needs to be assessed urgently.

We are only now beginning to understand the full spectrum of neurological syndromes associated with both Zika and chikungunya infections. For example, patient 19 showed clinical signs of myeloradiculitis and had neurophysiological evidence of AIDP, which is consistent with simultaneous diagnoses of both myelopathy and a form of GBS. Another patient (15) had an unusual combination of encephalitis, cerebral microangiopathy demonstrated on MRI and inflammatory myopathy based on neurophysiological studies. We saw evidence of extensive intramedullary myelitis in some patients, and anterior myelitis in another (e.g. patients 2 and 9 respectively, Fig 3); the latter is consistent with anterior horn cell disease seen in other flavivirus infections.[31] In the French Polynesian study, neurophysiological investigations showed that all Zika patients with GBS had the AMAN subtype;[4] whereas in the Colombian study, almost all had AIDP.[5] In our study we additionally found AMSAN, confirming involvement of the sensory axons in Zika virus-associated GBS. One patient with AMSAN had anti-GD1a antibodies, which is more normally associated with AMAN. Anti-GD1a antibodies were also detected in Zika and chikungunya patients with central nervous system disease (Table 2). We also found anti-GM1, anti-GD1b, and anti-GQ1b antibodies in patients with central nervous system disease. Anti-GM1 antibodies are associated with AMAN, anti-GD1b with sensory ataxic neuropathy, and anti-GQ1b with Miller Fisher syndrome and Bickerstaff’s brainstem encephalitis.[32] The significance of detecting these antibodies in patients with encephalitis and myelitis is not certain.

Although in our study six patients had evidence of recent systemic dengue virus infection, five of them also had evidence of CNS infection with a different virus, suggesting dengue on its own was less likely to be the cause of the neurological disease. We might have expected to see more dengue-associated neurology, given that the virus is circulating widely in Rio de Janeiro[33] and is a well-recognised cause of neurological disease.[22] Whether this in some way reflects the fact that it has been circulating for over 30 years in this city,[34] but Zika and chikungunya viruses have been newly introduced, is not known. Alternatively, this may be due to differing neurovirulence of the three viruses.

For dengue, secondary infection is a risk factor for more severe systemic dengue disease, a phenomenon thought to be mediated by antibody-dependent enhancement.[35] Interestingly, in a recent in vitro study, plasma immune to dengue virus induced potent antibody-dependent enhancement of Zika virus.[36] In our cases, all serum samples were positive for dengue IgG antibody, indicating prior flavivirus exposure, a pattern also seen in 86% of tested patients in the Colombian report on GBS.[5] Larger prospective studies are needed to investigate whether such dengue exposure is a risk factor for developing neurological disease after Zika virus infection.

Combined infection of arboviruses has not been well described in those with neurological presentations. In our patients with evidence of dual infections, whether the neurological disease was caused by one arbovirus or the other, or by a combination of the two is unclear. The fact that so many of our patients had evidence of dual infection may indicate that combined infections are responsible for severe disease, as we have seen in other settings.[37]

Flaviviruses can cause neurological disease by attacking the nervous system directly or indirectly via immune-mediated processes; the latter tend to occur some time after the acute infection, making virological diagnosis especially challenging. Detection of virus in the CSF is usually taken as the strongest evidence of causality, but it has often cleared by the time patients present, making us reliant on detection of virus systemically or demonstration of CSF or serum IgM antibody. For both Zika and chikungunya, whether testing urine for RNA increases the window of detection compared to serum is debated.[38–40] In our series, six patients (two with CNS disease) with no virus in the CSF or serum had virus detected in the urine (five Zika, one chikungunya), underscoring the value of testing this sample.

Our results must be interpreted in the context of the study’s limitations. First, cross-reactivity between flaviviruses makes distinguishing Zika from dengue by serological tests challenging.[4, 8] In four patients (two of whom were positive for Zika by PCR), we found elevated IgM antibody to both dengue and Zika, which may represent cross reactivity. Even in cases where CSF IgM was detected for Zika but not dengue virus, given the unknown specificity and sensitivity of the available flavivirus serological tests, caution must be applied. Newer assays in development and plaque reduction neutralization testing will help in the future. Differentiating infections clinically was also difficult; conjunctivitis is commonly seen in Zika[1] and not often reported for dengue or chikungunya, but we did not find conjunctivitis or any other clinical features that could distinguish the infections in our series. We did not look for West Nile virus because it was not circulating in Rio de Janeiro at the time of our study, but this may be important in other settings. Second, given the retrospective nature of the study, we did not have all CSF, serum and urine samples for each patient, thus potentially under-diagnosing arboviral infections in the cohort; in addition, the timing of sample collection was not standardised. Third, we only studied patients who had symptoms consistent with Zika infection. Whether Zika virus can case neurological disease in patients with no febrile illness will need to be addressed in future studies. Fourth, our study included a relatively small number of patients, thus the spectrum of neurology and role of chikungunya described may be even more extensive.

In summary, our study adds to the growing body of evidence arguing for a wide spectrum of neurological disease associated with Zika virus infection, including central nervous system disease. Some patients in whom a Zika virus-associated neurological disorder was suspected were actually infected with chikungunya virus, and many were infected with more than one arbovirus. The Zika public health emergency was recently declared over, recognising that the virus is here to stay and a sustained technical and research response is needed.[7] To understand fully the disease burden of Zika virus, clinicians and public health officials need to look beyond GBS, and also to investigate for other arboviruses that may cause similar neurological disease, particularly chikungunya.

## Supporting information

S1 Checklist STROBE checklist. (DOC)

S1 Appendix Additional information including hospital names, diagnostic criteria, diagnoses of patients without evidence of arbovirus infection, neurophysiology data, immunological assays and days between infection and sample collection, and statistical analyses. (DOCX)
