# Correction: The spectrum of neurological disease associated with Zika and chikungunya viruses in adults in Rio de Janeiro, Brazil: A case series

**DOI:** 10.1371/journal.pntd.0007166

**Published:** 2019-01-31

**Authors:** The PLOS Neglected Tropical Diseases Staff

## Notice of Republication

This article was republished on January 2, 2019, to correct errors to Box 1 introduced during the typesetting process. The publisher apologizes for the errors. Please download this article again to view the correct version.
