# Supplementary material for: The spectrum of neurological disease associated with Zika and chikungunya viruses in adults in Rio de Janeiro, Brazil: A case series
Source: PLoS Negl Trop Dis. 2018 Feb 12;12(2):e0006212. doi: 10.1371/journal.pntd.0006212 (PMC5837186; doi:10.1371/journal.pntd.0006212)
Supplement: S1 Appendix — (DOCX) [file pntd.0006212.s002.docx]

**S1 Appendix**

**Supplement to: Mehta R, Soares C, Medialdea-Carrera R et al. The spectrum of neurological disease associated with Zika and chikungunya viruses in adults in Rio de Janeiro, Brazil.**

**Table of contents:**

| **Page(s)** | **Content** |
| --- | --- |
| 2 | Table of contents |
| 3 | Hospitals |
| 4-7 | Diagnostic criteria |
| 8 | Table 1 – diagnoses of patients without without evidence for a recent Zika, chikungunya or dengue virus infection |
| 9 | Table 2 – neurophysiology studies |
| 10-11 | Table 3 – serology levels and days between infection and sample collection for 22 patients presenting with arboviral-associated neurological disease |
| 12 | Statistical tables |
| 13 | References |

**Hospitals:**

Hospital Federal dos Servidores do Estado

Hospital Universitário Pedro Ernesto

Instituto de Pesquisa Clínica Evandro Chagas (IPEC)

Hospital do Andaraí

Hospital Geral de Bonsucesso

Hospital Barra D'or

Hospital de Clínicas de Niterói

Hospital Bangu D'Or

Hospital Icaraí

Hospital Badim

Hospital São Vicente de Paulo

**Diagnostic criteria:**

1. Guillain-Barré syndrome (adapted from Sejvar et al. 2011, Hadden et al. 1998)[1, 2]

| **Level 1** | **Level 2** | **Level 3** | **Level 4** |
| --- | --- | --- | --- |
| [ ] Bilateral and flaccid weakness of the limbs  **AND**  [ ] Absence of an alternative diagnosis for weakness  **AND**  [ ] Decreased or absent deep tendon reflexes in affected limbs  **AND**  [ ] Monophasic illness pattern with weakness nadir between 12 hours and 28 days, followed by clinical plateau | | | Suspected GBS with no other diagnosis apparent, but does not fulfil level 3 criteria |
| [ ] CSF total white cell count < 50 cells/mm^3^  **OR**  [ ] If CSF results unavailable, electrophysiological findings consistent with GBS | |  |  |
|  |  |  |  |
| [ ] CSF protein level above laboratory normal value AND CSF total white cell count < 50 cells/mm^3^  **AND**  [ ] Electrophysiological findings consistent with GBS |  |  |  |
| **Classify 🡪 [ ] Level 1** | **[ ] Level 2** | **[ ] Level 3** | **[ ] Level 4** |

2. Encephalitis (adapted from Granerod et al. 2010, Venkatesan et al. 2013)[3, 4]

| **Level 1** | **Level 2** | | **Level 3** |
| --- | --- | --- | --- |
| [ ] Acute or sub acute (<4 weeks) alteration in consciousness, cognition, personality or behaviour persisting for more than 24 hours  [ ] Absence of an alternative diagnosis for symptoms | | | |
| [ ] New onset seizure  **OR**  [ ] New focal neurological signs  **OR**  [ ] Fever (≥ 38ºC)  **OR**  [ ] Movement disorder (includes: Parkinsonism, oromotor dysfunction etc.)  **OR**  [ ] EEG consistent with encephalopathy +/- epileptiform features | | |  |
| [ ] CSF total white cell count > 5 cells/mm^3^  **OR**  [ ] Neuroimaging compatible with encephalitis | |  | |
| **Classify 🡪 [ ] Level 1** | | **[ ] Level 2** | **[ ] Level 3** |

3. Myelitis (adapted from Transverse Myelitis Consortium Working Group Criteria, 2002.)[5]

| **Level 1** | **Level 2** | | **Level 3** | **Diagnosis unclear** |
| --- | --- | --- | --- | --- |
| [ ] Acute onset of weakness or sensory disturbance of upper and/or lower limbs  [ ] Absence of an alternative diagnosis for symptoms | | | | |
| [ ] Brisk reflexes or extensor plantar response  **OR**  [ ] Bladder or bowel dysfunction  **OR**  [ ] Clearly defined sensory level | | | |  |
| [ ] Absence of extra-axial compressive aetiology by neuroimaging (**MRI or CT myelography)**  **AND**  [ ] Absence of flow voids on the surface of the spinal cord suggestive of arteriovenous malformation **(MRI)** | | |  | |
| [ ] CSF total white cell count > 5 cells/mm^3^  **OR**  [ ] MRI changes consist with myelitis | |  | | |
| **Classify 🡪[ ]Level 1** | | **[ ] Level 2** | **[ ] Level 3** | **[ ] Diagnosis unclear** |

4. Meningitis (adapted from McGill F, Heyderman RS, Michael BD, *et al.* 2016)[6]

| **Level 1** | **Level 2** | **Level 3** |
| --- | --- | --- |
| [ ] Absence of an alternative diagnosis for symptoms  **AND**  [ ] Neck stiffness  **OR**  [ ] Kernig’s sign positive (with the hip and knee at 90° angles, subsequent extension of the knee causes pain)  **OR**  [ ] Brudzinsky’s sign positive (involuntary lifting of the legs on lifting the patient's head from the examining couch) | | |
| [ ] Fever (≥ 38ºC) | |  |
| [ ] CSF total white cell count > 5 cells/mm^3^  **OR**  [ ] Meningeal enhancement seen on contrast enhanced CT or MRI |  | |
| **Classify 🡪 [ ] Level 1** | **[ ] Level 2** | **[ ] Level 3** |

5. Acute Disseminated Encephalomyelitis (ADEM) - adapted from the clinical definition proposed by Krupp, Banwell, *et al.,* 2007[7]

6. Radiculitis - based on clinical features and electrophysiological studies[8]

7. Myositis - based on clinical features and electrophysiological studies

8. Facial diplegia with paraesthesia (GBS variant) - modified from Dimachkie & Barohn, 2013[9]

**Table 1 - Diagnoses of patients without evidence for a recent Zika, chikungunya or dengue virus infection (n=13):**

| **Diagnosis** | **Number of patients** |
| --- | --- |
| Guillain-Barré Syndrome | 5 |
| Miller Fisher Syndrome | 1 |
| Neuromyelitis optica | 1 |
| Encephalitis | 1 |
| Facial nerve palsy | 1 |
| Myeloradiculitis | 1 |
| Myelitis | 1 |
| Encephalomyeloradiculitis | 1 |
| Radicular pain | 1 |

**Table 2 – Neurophysiology studies for patients with abnormal neurophysiology, compound muscle action potentials:**

| **Patient number** | | **7** | | **12** | | **15** | | **16** | | **17** | | **18** | | **19** | | **21** | | |
| --- | --- | --- | --- | --- | --- | --- | --- | --- | --- | --- | --- | --- | --- | --- | --- | --- | --- | --- |
| **Neurophysiological diagnosis** | | **AMSAN** | | **AMSAN** | | **Inflammatory myopathy** | | **AMSAN** | | **AMAN** | | **AMSAN** | | **AIDP** | | **AMSAN** | | |
| **Clinical diagnosis** | | **GBS** | | **Myelo-radiculitis** | | **Encephalitis + myelitis** | | **Myelo-radiculitis** | | **GBS** | | **Myelo-radiculitis** | | **Myelo-radiculitis** | | **GBS** | | |
| **Right/Left** | | **R** | **L** | **R** | **L** | **R** | **L** | **R** | **L** | **R** | **L** | **R** | **L** | **R** | **L** | **R** | **L** |  |
| **MOTOR** | | | | | | | | | | | | | | | | | |  |
| MEDIAN | Amp | 2.8 | 7.1 | 4.2 | 3.6 | Data unavailable | | 7.4 | 11.1 | 15.2 | 15 | 2.5 | 3.4 | 7.18 | 9.2 | abs | 0.8 |  |
|  | DML | 5.3 | 0.9 | 3.5 | 3.8 |  |  | 3.8 | 3.7 | 3.3 | 3.2 | 4 | 2.8 | 4.9 | 4.5 | abs | 5 |  |
|  | CV | 38.2 | 44.8 | 47 | - |  |  | 53.8 | 55.9 | 57.7 | 51.6 | 52.4 | 50.1 | 46.4 | 49 | abs | 44 |  |
| ULNAR | Amp | 4.4 | - | 3.7 | 3.7 |  |  | 8 | 9.7 | 11.1 | 10.5 | 3.5 | 5.1 | 10.9 | 11 | abs | 0.6 |  |
|  | DML | 2.8 | - | 2.5 | 3.2 |  |  | 2.7 | 2.3 | 2.5 | 2.3 | 2.7 | 2.7 | 3.8 | 3.6 | abs | 2.9 |  |
|  | CV | 47.2 | - | 50.8 | 52.6 |  |  | 46.7 | 54.1 | 61.9 | 60 | 59.5 | 63.3 | 57.4 | 51 | abs | 43.9 |  |
| PERONEAL | Amp | abs | 4.3 | 0.5 | 0.7 |  |  | 1.3 | 10.6 | 1.9 | 1.4 | abs | abs | 7.5 | 1.35 | abs | abs |  |
|  | DML | abs | 8.3 | 5.9 | 5.8 |  |  | 4.8 | 4.7 | 5.2 | 5.6 | abs | abs | 4.7 | 7.4 | abs | abs |  |
|  | CV | abs | 39 | 43.7 | 47.6 |  |  | 36.7 | 37 | 39.8 | 38.8 | abs | abs | 35.8 | 41.5 | abs | abs |  |
| TIBIAL | Amp | 2 | 0.6 | 2.4 | - |  |  | 2.1 | 0.8 | 3 | 3.3 | - | abs | 13.4 | 12.4 | 0.6 | 0.7 |  |
|  | DML | 6.4 | 10.6 | 4.7 | - |  |  |  | 7.5 | 4.7 | 4.9 | - | abs | 6 | 6 | 6.8 | 7.5 |  |
|  | CV | 33 | 35 | 51.2 | - |  |  |  | 34.4 | 31.6 | 38.8 | - | abs | 37.3 | 40.2 | 34.5 | 40.6 |  |
| **SENSORY** | | | | | | | | | | | | | | | | | |  |
| MEDIAN | Amp | abs | abs | abs | 9.1 |  | | abs | - | 30.7 | 46.3 | 2.2 | 6.2 | 3.0 | 5.2 | abs | abs |  |
|  | Onset lat | abs | abs | abs | 2.4 |  |  | abs | - | 2.7 | 2.7 | 2.8 | 2.5 | 3.88 | 3.66 | abs | abs |  |
|  | CV | abs | abs | abs | 50.6 |  |  | abs | - | 57 | 55.1 | 45.8 | 55.3 | 43.8 | 41 | abs | abs |  |
| ULNAR | Amp | 22.3 | - | abs | abs |  |  | 7.7 | 11.9 | 29.4 | 31.9 | 11.7 | 3.5 | 2.2 | 5.2 | abs | abs |  |
|  | Onset lat | 1.9 | - | abs | abs |  |  | 2.5 | 2.2 | 2.3 | 2.2 | 2 | 2.2 | 2.94 | 2.8 | abs | abs |  |
|  | CV | 56.7 | - | abs | abs |  |  | 45.6 | 50.5 | 51.7 | 55.6 | 54.5 | 53.6 | 40.8 | 42.6 | abs | abs |  |
| SUPERFICIAL PERONEAL | Amp | 25.1 | 9 | - | - |  |  | - | - | 5.7 | 11.6 | abs | - | 13.9 | 10.7 | abs | abs |  |
|  | Peak lat | 4.2 | 4.1 | - | - |  |  | - | - | 3.2 | 3.5 | abs | - | 3.3 | 2.7 | abs | abs |  |
|  | CV | 48.4 | 43.7 | - | - |  |  | - | - | 61.9 | 56.5 | abs | - | 36.4 | 43.2 | abs | abs |  |
| SURAL | Amp | - | - | abs | 1 |  |  | 7.5 | 3.3 | - | - | abs | - | 8 | 12.2 | - | - |  |
|  | Peak lat | - | - | abs | 2.2 |  |  | 5.8 | 3.3 | - | - | abs | - | 4.4 | 3.5 | - | - |  |
|  | CV | - | - | abs | 63.3 |  |  | 27.4 | 42.8 | - | - | abs | - | 31.5 | 39.3 | - | - |  |

**Key:**

Amp=distal amplitude, DML=distal motor latency, CV=conduction velocity, abs=absent, "-"=test not done, red=abnormal result**Table 3 – Immunological assays and days between infection and sample collection for 22 patients presenting with arboviral-associated neurological disease, ordered by date of admission:**

| **Patient** | **Days between infection and sample collection** | | | **Zika** | | | | **Chikungunya** | | | | **Dengue** | | | |
| --- | --- | --- | --- | --- | --- | --- | --- | --- | --- | --- | --- | --- | --- | --- | --- |
|  | **CSF** | **Serum** | **Urine** | **CSF IgM**  **(P/N)** | **CSF IgG (RU/ml)** | **Serum IgM (Ratio)** | **Serum IgG**  **(RU/ml)** | **CSF IgM**  **(Ratio)** | **CSF IgG**  **(RU/ml)** | **Serum IgM**  **(Ratio)** | **Serum IgG (RU/ml)** | **CSF IgM**  **(PU)** | **CSF IgG**  **(PU)** | **Serum IgM**  **(PU)** | **Serum IgG**  **(PU)** |
| 1 | 11 | 11 | 11 | 5·5 | - | - | 111·5 | - | - | - | - | 21·1 | 63·2 | 23·8 | 60·5 |
| 2 | 25 | 25 | 25 | 35·6 | - | - | >200 | - | - | 1·2 | - | 17·7 | 16·0 | 43·6 | 62·7 |
| 3 | 4 | 6 | 4 | 7·1 | - | - | >200 | - | - | - | - | - | 21·8 | 13·7 | 66·2 |
| 4 | 20 | 20 | na | 3·1 | 40·3 | - | >200 | - | - | - | - | - | 15·1 | - | 69·4 |
| 5 | 23 | 20 | 20 | 27·7 | - | - | >200 | - | - | - | - | - | na | - | 60·5 |
| 6 | na | 33 | 33 | na | na | - | >200 | na | na | 1·2 | 124·0 | na | na | 47·7 | 65·0 |
| 7 | 18 | 18 | 19 | 41·1 | 369·3 | 1·2 | 55·4 | - | - | - | - | - | 21·6 | 12·2 | 62·3 |
| 8 | 29 | 29 | 29 | - | - | - | >200 | - | - | - | - | - | - | - | 57·1 |
| 9 | 10 | na | na | 8·7 | 155·8 | na | na | 2·5 | 92·0 | na | na | - | - | na | na |
| 10 | 15 | 15 | 70 | 25·1 | na | 1·5 | na | - | na | - | - | na | na | - | 55·2 |
| 11 | 26 | 26 | 26 | - | - | - | 114·3 | 1·1 | - | 1·6 | - | - | 32·6 | - | 57·7 |
| 12 | 12 | na | 12 | - | - | na | na | 3·1 | 75·8 | na | na | - | 23·8 | na | na |
| 13 | 6 | 9 | 9 | - | - | - | 40·6 | - | na | 6·9 | 73·2 | - | na | - | 60·0 |
| 14 | 31 | na | na | - | - | na | na | - | - | na | na | - | - | na | na |
| 15 | 7 | na | na | - | - | na | na | - | - | na | na | - | - | na | na |
| 16 | 23 | na | 23 | 3·5 | 67·5 | na | na | 3·0 | 56·3 | na | na | - | 17·1 | na | na |
| 17 | na | 71 | 72 | na | na | - | 22·0 | na | na | - | - | na | na | - | 59·3 |
| 18 | 23 | na | 23 | - | 37·2 | na | - | 1·5 | 35·9 | na | na | - | 16·0 | na | na |
| 19 | 13 | 13 | 13 | - | 151·6 | - | >200 | 1·8 | 58·4 | 4·4 | 112·5 | - | 25·3 | 14·1 | 64·5 |
| 20 | 35 | 25 | 36 | - | - | - | - | - | - | 6·8 | 89·2 | - | - | - | 53·6 |
| 21 | 72 | 72 | 72 | - | - | - | 86·4 | - | - | 2·1 | - | - | - | - | 54·3 |
| 22 | 66 | 60 | 60 | 4·5 | - | - | 24·6 | - | - | - | - | - | - | - | 65·7 |

Key:

"na" =sample not available/not enough volume; "-" =negative; CSF=cerebrospinal fluid. Units and cut-off values: Zika CSF IgM (P/N = ratio of patient optical density to negative control value); Pos: P/N >3. Zika serum IgM and chikungunya IgM (ratio = patient sample/cut-off value); Pos: ratio ≥1·1. Zika IgG and Chikungunya IgG (relative units/ml, RU/ml as described by the manufacturer); Pos: RU/ml ≥22. Dengue IgM and IgG (Panbio Units, PU = ratio of patient optical density to cut-off value x10); Pos: PU >11.

**Statistical analyses:**

Table A: Wilcoxon-Mann-Whitney U-test comparing median time from infection to neurology between CNS and peripheral nervous system disease

|  | Median time from infection to neurology (days) | P-value |
| --- | --- | --- |
| CNS disease +/- peripheral disease  (n=14) | 4 | 0.009 |
| Peripheral nervous system disease only  (n=6) | 11 |  |

Table B: Wilcoxon-Mann-Whitney U-test comparing median time from infection to neurology between CNS disease (+/- peripheral disease) associated with and without CNS Zika

|  | Median time from infection to neurology (days) | P-value |
| --- | --- | --- |
| CNS disease associated with CNS Zika (n=5) | 1 | 0.216 |
| CNS disease not associated with CNS Zika  (n=9) | 4 |  |

Table C: Wilcoxon-Mann-Whitney U-test comparing median time from infection to neurology between peripheral nervous system disease only associated with and without CNS Zika

|  | Median time from infection to neurology (days) | P-value |
| --- | --- | --- |
| Peripheral nervous sytstem disease only associated with CNS Zika  (n=3) | 10 | 0.400 |
| Peripheral nervous system disease only not associated with CNS Zika  (n=3) | 29 |  |

**References**

1. Sejvar JJ, Kohl KS, Gidudu J, Amato A, Bakshi N, Baxter R, et al. Guillain-Barre syndrome and Fisher syndrome: case definitions and guidelines for collection, analysis, and presentation of immunization safety data. Vaccine. 2010;29(3):599-612.

2. Hadden RD, Cornblath DR, Hughes RA, Zielasek J, Hartung HP, Toyka KV, et al. Electrophysiological classification of Guillain-Barre syndrome: clinical associations and outcome. Plasma Exchange/Sandoglobulin Guillain-Barre Syndrome Trial Group. Ann Neurol. 1998;44(5):780-8.

3. Granerod J, Ambrose HE, Davies NW, Clewley JP, Walsh AL, Morgan D, et al. Causes of encephalitis and differences in their clinical presentations in England: a multicentre, population-based prospective study. Lancet Infect Dis. 2010;10(12):835-44.

4. Venkatesan A, Tunkel AR, Bloch KC, Lauring AS, Sejvar J, Bitnun A, et al. Case definitions, diagnostic algorithms, and priorities in encephalitis: consensus statement of the international encephalitis consortium. Clin Infect Dis. 2013;57(8):1114-28.

5. Transverse Myelitis Consortium Working Group. Proposed diagnostic criteria and nosology of acute transverse myelitis. Neurology. 2002;59(4):499-505.

6. McGill F, Heyderman RS, Michael BD, Defres S, Beeching NJ, Borrow R, et al. The UK joint specialist societies guideline on the diagnosis and management of acute meningitis and meningococcal sepsis in immunocompetent adults. J Infect. 2016;72(4):405-38.

7. Krupp LB, Banwell B, Tenembaum S. Consensus definitions proposed for pediatric multiple sclerosis and related disorders. Neurology. 2007;68(16 Suppl 2):S7-12.

8. Wilbourn AJ, Aminoff MJ. AAEM minimonograph 32: the electrodiagnostic examination in patients with radiculopathies. American Association of Electrodiagnostic Medicine. Muscle Nerve. 1998;21(12):1612-31.

9. Dimachkie MM, Barohn RJ. Guillain-Barre syndrome and variants. Neurol Clin. 2013;31(2):491-510.
